# BASCO: a toolbox for task-related functional connectivity

**DOI:** 10.3389/fnsys.2015.00126

**Published:** 2015-09-10

**Authors:** Martin Göttlich, Frederike Beyer, Ulrike M. Krämer

**Affiliations:** ^1^Department of Neurology, University of LübeckLübeck, Germany; ^2^Institute for Psychology II, University of LübeckLübeck, Germany

**Keywords:** fMRI, functional connectivity, network analysis, beta-series correlation, degree centrality, event-related design

## Abstract

BASCO (BetA Series COrrelation) is a user-friendly MATLAB toolbox with a graphical user interface (GUI) which allows investigating functional connectivity in event-related functional magnetic resonance imaging (fMRI) data. Connectivity analyses extend and compliment univariate activation analyses since the actual interaction between brain regions involved in a task can be explored. BASCO supports seed-based functional connectivity as well as brain network analyses. Although there are a multitude of advanced toolboxes for investigating resting-state functional connectivity, BASCO is the first toolbox for evaluating task-related whole-brain functional connectivity employing a large number of network nodes. Thus, BASCO allows investigating task-specific rather than resting-state networks. Here, we summarize the main features of the toolbox and describe the methods and algorithms.

## Introduction

BASCO (BetA Series COrrelation) is a Matlab® toolbox for investigating inter-regional functional connectivity in event-related fMRI data. The toolbox is best suited for slow event-related designs with trial duration longer than the repetition time (TR). Deriving the functional connectivity from event-related fMRI data using beta-series correlation complements other approaches to investigate brain connectivity and network integration. Generally one distinguishes between structural, functional and effective connectivity. Structural (or anatomical) connectivity can be non-invasively assessed by white matter fiber tracking using diffusion tensor imaging (DTI). This method is frequently used in connectome analyses investigating network topology (Sporns et al., 2004). Functional connectivity expresses the statistical dependency among activations in different brain regions and results in undirected (symmetrical) connectivity matrices. Effective connectivity describes the influence that one brain region exerts over another and leads to directed (asymmetrical) connectivity matrices. For instance, psychophysiological Interaction (PPI) analyses allow exploring how brain activity is modulated by one source region and one experimental factor (Friston et al., 1997). Strong modulatory influence of the source region on a given target region can be interpreted as strong (directed) connectivity. Dynamic Causal Modeling (DCM) (Friston et al., 2003), another method to investigate effective connectivity, allows comparing a set of a priori models describing a dynamical system and selects the best model generating the data.

While functional connectivity just explores the correlation between brain activity in different brain regions, effective connectivity establishes a causal relationship and thus conveys deeper insights into brain network organization. The strength of the functional connectivity analyses implemented in BASCO, however, lies in the possibility to explore brain networks with a large number of nodes. Even voxel-wise network analyses are feasible as will be demonstrated below. In contrast to PPI and DCM analyses, this approach thus allows performing data-driven network analyses without specifying a priori seed-regions or a set of models describing the interaction between brain regions.

A common approach to investigate seed-based functional connectivity and whole-brain network organization is based on resting-state fMRI data (Biswal et al., 1995; Beckmann et al., 2005; Fox et al., 2005; De Luca et al., 2006; Fox and Raichle, 2007; Buckner et al., 2008; Biswal et al., 2010; van den Heuvel and Hulshoff Pol, 2010). Inter-regional functional connectivity is derived from the temporal correlation of low frequency BOLD fluctuations (< 0.1 Hz) when subjects are not engaged in any specific task. Seed-based analyses (Biswal et al., 1995), graph theoretical approaches (van den Heuvel and Hulshoff Pol, 2010) and approaches using independent component analyses (ICA) (Beckmann et al., 2005; De Luca et al., 2006) of resting-state data revealed distinct networks of coherent spontaneous BOLD fluctuations reflecting the functional architecture of the human brain. Resting-state analyses are frequently used in clinical populations to identify disease-related abnormalities in functional connectivity and to gain new insights into the neural correlates of neurodegenerative or psychiatric diseases. Resting-state fMRI is well suited for clinical populations as it is less demanding in terms of compliance compared to task-based fMRI and also applicable to strongly affected groups as patients with Alzheimer's disease (Greicius et al., 2004; Allen et al., 2007; Brier et al., 2012) or disorders of consciousness (Soddu et al., 2011; Crone et al., 2014).

Although resting-state connectivity differences are often discussed in context of task-related changes or group differences, it is unclear to what extent changes in resting-state connectivity can be translated to differences in task-related brain activity. BASCO thus focusses on functional connectivity patterns related to specific experimental manipulations. This allows investigating task-related data from a network perspective using graph theory and beta-series correlations. For instance, the dissociation into different network modules can be studied and condition-specific provincial as well as connector hubs important for network integration can be identified. Provincial hubs are network nodes which are densely connected within their module, whereas connector hubs play a crucial role connecting different network modules (Bullmore and Sporns, 2009; Rubinov and Sporns, 2010; Sporns, 2013a,b). This underlines the advantage of a network approach as regions similarly modulated by task conditions can be dissociated into distinct networks and regions crucial for network integration can be identified.

In this article we first give an overview of all analysis approaches which are provided by the BASCO toolbox. Then we introduce the methods which are implemented in the toolbox. For illustration, we present data for each of the analysis approaches. The article closes with a discussion.

## Overview of toolbox functions

**Figure 1:**
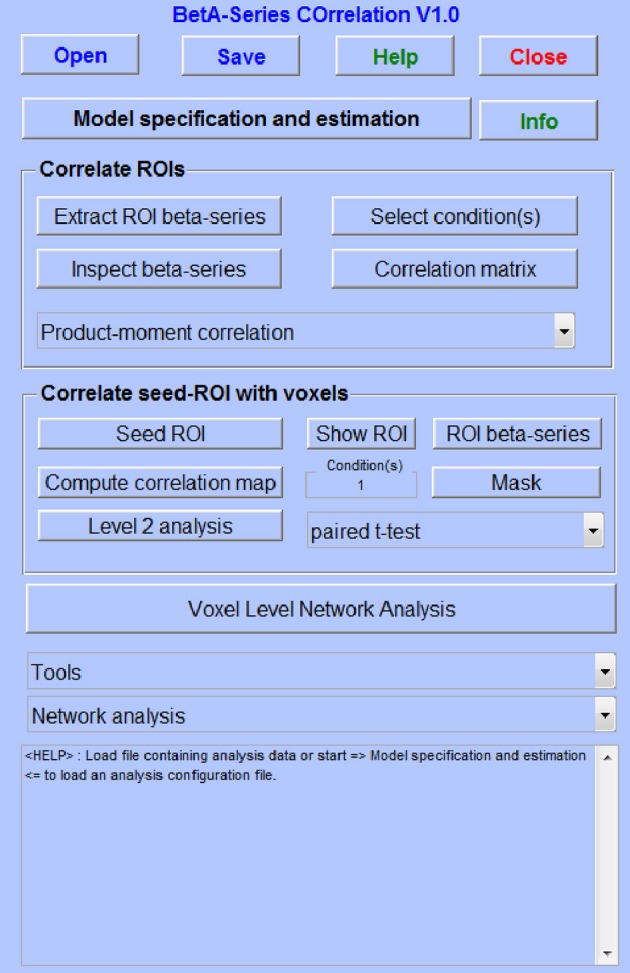
**The BASCO GUI**.

The BASCO toolbox provides a user-friendly graphical user interface (GUI) (Figure 1). It is based on an approach introduced by Rissman et al. (2004) and frequently used in task-based network analyses (Ye et al., 2011; Brunnlieb et al., 2013). In contrast to a standard univariate analysis, a general linear model (GLM) is fitted to the data where the evoked activity in each trial is modeled by a separate covariate. This results in a series of beta-values for each voxel which is related to a given experimental condition. The functional connectivity between brain regions is derived from correlating beta-series. Estimated movement parameters can be included in the GLM. The parameter estimation is performed using SPM. BASCO offers the following analysis approaches:

- Seed-based functional connectivity analysis. This yields functional connectivity maps using a defined seed-region. The mean beta-series is estimated for the seed-region and correlated to the beta-series of each individual voxel within the brain.
- ROI-based network analysis. Given a parcellation of the brain, e.g., based on the AAL atlas (Tzourio-Mazoyer et al., 2002), the mean beta-series are extracted for each ROI and a network matrix is calculated correlating all ROI beta-series.
- Voxel-based whole brain network analysis. BASCO allows creating voxel-wise degree centrality maps for a selected experimental condition. The degree centrality of a voxel is defined as the number of connections to other voxels in the brain which are stronger than a defined absolute threshold. The degree centrality is a sensitive marker for functional connectivity with high spatial resolution.

Connectivity analyses are performed for a specific condition or a set of conditions on individual subjects. The modulation of functional connectivity by an experimental condition can be investigated on group-level using a paired *t*-test. Between-group effects can be studied performing a two-sample *t*-test using connectivity maps for the same experimental condition.

The toolbox offers several functions to explore the data quality, e.g., detection of artifacts, review of the GLM fit, inspection of the beta-series and of the correlation matrices. It also offers tools to test for between-group or between-condition effects in functional connectivity and to perform a Graph analysis on the basis of network matrices (ROI-based analysis). It allows visualizing the data using the BrainNet Viewer (http://www.nitrc.org/projects/bnv/) or CIRCOS (Krzywinski et al., 2009).

BASCO is a Matlab® toolbox (R2009b or higher) and depends on SPM (http://www.fil.ion.ucl.ac.uk/spm/) and Marsbar (http://marsbar.sourceforge.net/; version 0.44 or higher). It is compatible with SPM8 and SPM12 and was tested on Linux and Windows systems. The Brain Connectivity Toolbox (BCT, http://www.brain-connectivity-toolbox.net/) is required for network analyses. BASCO is publicly available under the GNU General Public License (GPL) at http://www.nitrc.org/projects/basco. A manual and tutorial data can be downloaded from the NITRC (Neuro-Informatics Tools and Resources) project pages.

## Methods implemented in BASCO

### Beta-series correlation

The functional connectivity between brain regions is established by a so-called beta-series correlation analysis. This approach was first introduced by Rissman et al. (2004) and allows investigating inter-regional functional connectivity in event-related fMRI data. The method is implemented on the basis of a GLM where the evoked brain activity in each trial is modeled by a separate covariate time-locked to the stimulus onset. The GLM approach can be expressed as:

$$\begin{array}{l} Y=X\beta +\;\epsilon \end{array}$$

Here, *Y* denotes the measurement (the BOLD signal; n measurements), β is a vector containing the parameters that are to be estimated (m parameters), *X* the design matrix (the neural model; n × m matrix), and ε is the error term (n entries), which describes the deviation of the fitted model to the actual measurement. Estimating the model using a single regressor for each trial renders a series of beta-values for each voxel which is related to a given experimental condition. Estimated movement parameters are included in the GLM and the classical parameter estimation is performed using SPM (Wellcome Trust Centre for Neuroimaging, London; http://www.fil.ion.ucl.ac.uk/spm). Brain activity during each trial is modeled using the canonical hemodynamic response function (HRF). By default, the design matrix also includes the six motion regressors (x, y, z, pitch, roll, yaw) estimated in the motion correction step during the preprocessing to minimize signal-correlated motion effects. A high-pass filter of 128 s (default setting) is applied to the data. Classical parameter estimation is performed with a one-lag autoregressive model AR(1) to account for serial correlations in fMRI time series due to aliased biorhythms and un-modeled neuronal activity. This approach is best suited for slow event-related designs with trial duration longer than the repetition time (TR).

Hemodynamic derivative terms can be incorporated into the model to deal with variable hemodynamic delays and variable durations of brain activity (Friston et al., 1998). This results in three beta-maps per regressor. Following the approach proposed by Calhoun et al. (2004) the beta-maps are combined to derive an “amplitude of effect” map using the following formula:

$$\begin{array}{l} \hat{\beta }=sign(\beta_{1})\sqrt{\beta_{1}^{2}+\beta_{2}^{2}+\beta_{3}^{2}} \end{array}$$

Here, the first beta-value relates to the canonical HRF, whereas the second and third beta-values relate to the first and second temporal derivative, respectively. The sign-function returns +1 or -1 depending on the sign of the argument. Following this approach, a single value for each voxel and each trial is derived from the three beta-values. This “amplitude of effect” value is used in the correlation analysis.

The correlations between beta-series are evaluated using Pearson's linear correlation coefficient:

$$r=\frac{\sum_{i=1}^{N}\left(X_{i}-\bar{X}\right)\left(Y_{i}-\bar{Y}\right)}{\sqrt{\sum_{i=1}^{N}{\left(X_{i}-\bar{X}\right)}^{2}}\sqrt{\sum_{i=1}^{N}{\left(Y_{i}-\bar{Y}\right)}^{2}}}$$

*N* is the number of trials per condition and *X*_*i*_ and *Y*_*i*_ are the fitted beta-values (or “amplitude of effect” values) for trial *i*. *X* and *Y* are extracted for different voxels or brain regions. To allow for averaging and statistical testing, a Fisher z-transformation is applied to the sample correlation coefficient *r*:

$$\begin{array}{l} z=\frac{1}{2}ln\;\left(\frac{1+r}{1-r}\right) \end{array}$$

Here, “*ln*” is the natural logarithm. The Fisher z-transformed correlation coefficient is approximately normally distributed with a standard error given by

$$\begin{array}{l} \sigma =\frac{1}{\sqrt{N-3}} \end{array}$$

This formula is useful to judge the sensitivity of an experiment given an expected effect size.

### Seed-based connectivity analysis

The BASCO toolbox allows performing seed-based functional connectivity analyses, where the mean regional beta-series in a seed region is correlated with all voxel beta-series. This approach is useful in a hypothesis-driven analysis where a priori seed regions can be defined. The mean beta-series is calculated by averaging over all voxels comprising the seed region. This beta-series is then correlated with all voxels inside a user-defined mask or the whole brain. The resulting brain maps are Fisher z-transformed. The functional connectivity maps are saved as 3D NIfTI file (Neuroimaging Informatics Technology Initiative; http://nifti.nimh.nih.gov/) and can be used for group-level analyses.

### ROI-based network analysis

Given a parcellation of the brain, the mean beta-series is extracted for each ROI and a network matrix is calculated correlating all ROI beta-series with each other applying Pearson's linear correlation. Correlation coefficients are Fisher-z transformed to allow for averaging and statistical testing. BASCO also offers to assess the statistical dependence between beta-series using Spearman's rank correlation coefficient. Spearman correlation is less sensitive to strong outliers compared to the Pearson correlation and allows to test if two variables are monotonically related and is not restricted to linear relationships. BASCO allows performing data quality checks to spot outliers in the beta-series (see the BASCO manual for more details). If correlation coefficients are dominated by outliers it is more appropriate to use Spearman's correlation than Pearson correlation.

Connectivity matrices for each experimental condition can be obtained. BASCO offers a tool to test for significant differences in connectivity (edges) between-groups for the same condition (two-sample *t*-test) or within-group for different experimental conditions (paired *t*-test). Permutation test are also implemented. The tool also allows plotting the distribution of correlation coefficients to identify outliers and to check if the network weights are normally distributed. Let *n*_*N*_ be the number of network nodes, then the number of unique edges n_E_ in a symmetric connectivity matrix is given by:

$$\begin{array}{l} n_{E}=\frac{n_{N}(n_{N}-1)}{2} \end{array}$$

The number of network edges increases quadratically with the number of nodes. An FDR procedure is implemented to correct for multiple testing (Storey, 2002).

BASCO also offers a tool to calculate several network properties as the degree centrality, betweenness centrality, eigenvector centrality, clustering coefficient and the characteristic path length (Rubinov and Sporns, 2010). The BASCO release contains a script which demonstrates how to access the data written by BASCO to retrieve the network matrices and to perform an analysis of various network metrics. The user may also easily adapt the script to save the connectivity matrices in a format which can be accessed by other existing toolboxes to perform Graph analyses, e.g., GraphVar (Kruschwitz et al., 2015).

For convenience, several brain atlases are included in the toolbox. The data are available in a format which can be directly used in BASCO. (i) The Automatic Anatomical Labeling (AAL) atlas provides a parcellation of the brain into 116 regions (Tzourio-Mazoyer et al., 2002). This atlas is based on an anatomical parcellation according to major sulci and gyri using a spatially normalized single subject high resolution T1 volume provided by the Montreal Neurological Institute (MNI) (Collins et al., 1998). (ii) A parcellation of the brain divided into 160 regions of interest (ROIs) derived from a series of meta-analyses of task-related fMRI studies (Dosenbach et al., 2010). (iii) A brain atlas provided by Craddock et al. (2012) which was generated via spatially constrained spectral clustering. Craddock et al. (2012) offer several atlases which differ in their level of clustering, i.e., the number and size of the individual clusters. The atlases by Craddock et al. (2012) have been made publicly available at: http://www.nitrc.org/projects/cluster_roi/.

### Voxel-based network analysis

ROI-based network analyses depend on parcellation schemes which divide the brain into sub-regions. These brain regions may be rather large in terms of the number of voxels which they contain. This is problematic if the regions are not functionally homogeneous and the question remains whether the results of a network analysis depend on the applied parcellation scheme. Moreover, analyses using different parcellation schemes, i.e., different number of nodes, are not directly comparable. An approach to circumvent these problems is to use voxels as nodes of a network instead of larger regions. The advantages are that the results are not biased by the choice of the parcellation scheme and that a higher spatial resolution is obtained. However, due to the large number of nodes the calculation of network properties becomes very demanding as the number of correlations which have to be evaluated increase quadratically with the number of network nodes. BASCO offers to calculate degree centrality maps in a fast and efficient way. The degree centrality of a node is defined as the number of connections to other nodes in the network:

(7) $$D_{i}=\sum_{j=1}^{N}b_{ij}$$

Here, *D*_*i*_ denotes the degree of node *i, N* is the number of nodes and (*b*_*ij*_) is the binary connectivity matrix. BASCO requires the user to specify the experimental condition for which the connectivity analysis is performed, a mask including all voxels which are taken into account and an absolute threshold to binarize the weighted connectivity matrix. BASCO also calculates the node strength:

(8) $$S_{i}=\sum_{j=1}^{N}w_{ij}$$

Here, (*w*_*ij*_) denotes the thresholded connectivity matrix. The degree centrality and strength maps are saved as 3D NIfTI file and can be used for group-level analyses.

Calculating voxel-wise degree centrality maps involves a large number of voxels. For a whole-brain analysis and a typical voxel-size of 3 × 3 × 3 mm^3^ the number of voxels amounts to about *N* = 70,000. Holding the full correlation matrix in memory would require about 40 GB of RAM (double precision; 64-bit). We thus avoided keeping the full connectivity matrix in memory by computing the degree centrality for each voxel separately. In the following the algorithm is described in detail. Computation times refer to the following hardware and software configuration: Intel Core i5-2500 CPU @ 3.30 GHz; 32 GB RAM; OS: 64-bit Windows Professional; MATLAB R2012b benchmark: LU 0.05, FFT 0.08, and a relative speed of 90% with respect to a standardized hardware configuration. See the MATLAB documentation for more details on the benchmark. Let *A* be an *M* × *N* matrix containing the *M* beta-values for all *N* voxels. In a first step we standardized all columns of *A*:

(9) $$\begin{array}{l} Z_{ij}=\frac{A_{ij}-{\bar{A}}_{j}}{\sqrt{\sum_{i}^{M}{\left(A_{ij}-{\bar{A}}_{j}\right)}^{2}}}\\ {\bar{A}}_{j}=\frac{1}{M}\sum_{i}^{M}A_{ij} \end{array}$$

Here, *j* denotes the column, i.e., all *M* beta-values for a voxel *j*. Standardizing the measurement matrix *A* is done efficiently using the MATLAB function “bsxfun”:

$$\begin{array}{l} \text{An}=\text{bsxfun}(@\text{minus},\text{ A},\text{ mean}(\text{A},\text{ 1}));\\ \text{An}=\text{bsxfun}(@\text{times},\text{ An},\text{ 1}.\text{/sqrt}(\text{sum}(\text{An}.^{\wedge }\text{2},\text{ 1}))); \end{array}$$

For *N* = 70,000 voxels these calculations will take less than 20 ms. The correlation matrix can now be computed by a simple matrix multiplication, which is performed very efficiently within MATLAB: *C* = *Z*^*T*^*Z*. As stated above, we followed a different approach to minimize the memory consumption which is often a limiting factor to ensure that the algorithm runs on any hardware configuration and for analyses involving an even larger number of voxels. All correlation coefficients for a given voxel are computed in one step using the following MATLAB command:

$$\text{C = sum}(\text{repmat}(\text{An}(:,\text{j}),\text{ 1},\text{ Nvox}).^{*}\text{An},\text{ 1});$$

The correlation coefficients are Fisher z-transformed and a threshold is applied. The degree of voxel j is then computed by counting all correlation coefficients which are above the threshold. The computation time per degree centrality map (*M* = 24 beta-values; *N* = 70000 voxels) amounts to about 10 min on the platform described above.

Frequently, a different approach is proposed which takes advantage of the matrix product being associative:

$$\begin{array}{l} S=\left(Z^{T}Z\right)\vec{1}=Z^{T}(Z\vec{1}) \end{array}$$

Here, $\vec{1}$ is an N-dimensional vector containing only ones and *S* is an N-dimensional vector containing the sum of all correlation coefficients for each voxel. While this solution is extremely fast it is not accurate as negative (anti-correlations) and positive correlations should not be averaged and because correlation coefficient are not additive. This is why a Fisher z-transformation was applied in the preferred algorithm described above. Furthermore, this approach does not allow for thresholding. For some applications this approach might be useful and is thus also available.

## Illustration

**Figure 2:**
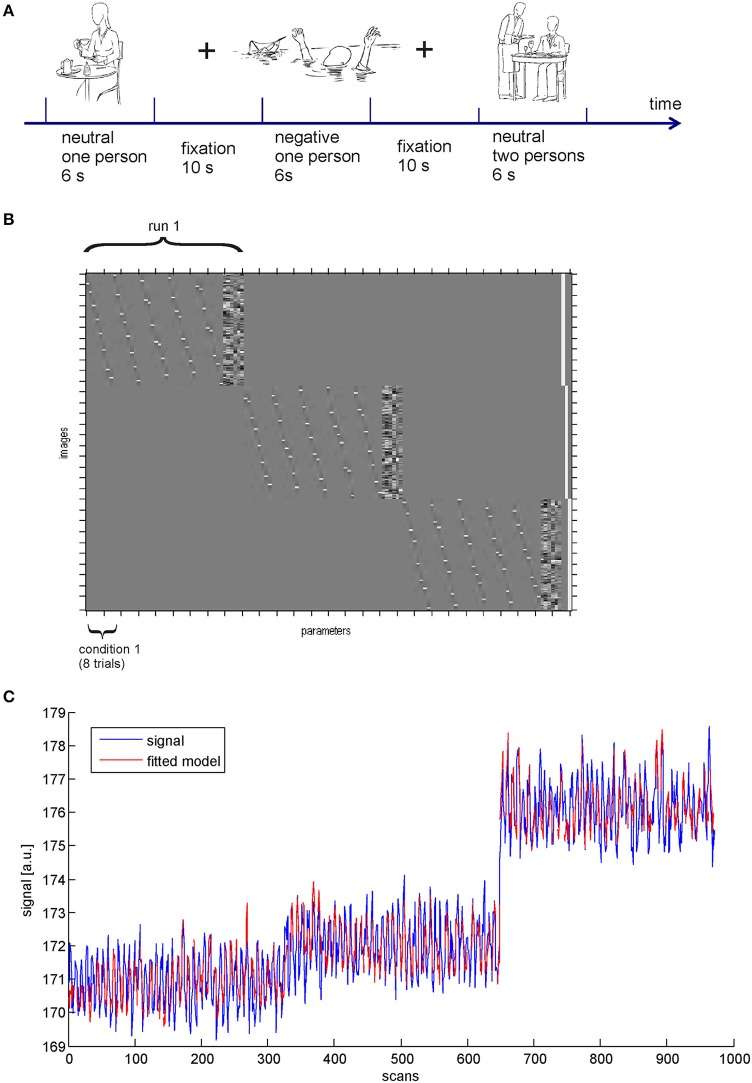
**(A)** Experimental design. **(B)** SPM design matrix. **(C)** GLM fit in the occipital cortex.

In the following, we illustrate the functionality of the BASCO toolbox using the tutorial data set available from the project pages. The tutorial data contains measurements from two different subjects who performed a paradigm employing social-emotional stimuli. The participants viewed line drawings depicting either one or two persons (social factor) in an emotionally neutral or negative scene (emotional factor). Three experimental runs employing a slow event-related design were conducted (Figure 2A). Pictures were presented in random order but with no more than two successive pictures of the same condition. Each picture was presented for 6 s, followed by a fixation cross (always 10 s). Each run comprised 40 drawing, eight per condition. The data was acquired using a 3-T Siemens Magnetom Trio scanner employing a single-shot gradient echo echo-planar imaging (EPI) sequence (repetition time 2 s). The data was preprocessed using SPM. Preprocessing included slice time correction, motion correction, spatial normalization and spatial smoothing (Gaussian kernel with 8 mm FWHM). The functional data was resampled to 3 × 3 × 3 mm^3^. For more details on the paradigm, the data acquisition and the preprocessing we refer to Krämer et al. (2010).

The SPM design matrix is shown in Figure 2B. In this example we followed exactly the same approach as Rissman et al. (2004), i.e., we did not include temporal derivatives of the HRF. In contrast to a standard univariate analysis of brain activity rather than connectivity the model was not estimated using a single regressor for all trials of a single experimental condition but a regressor for each individual trial was introduced. For each voxel this resulted in *n* = 24 beta-values per experimental condition. We obtained a reasonable model fit to the data as depicted in Figure 2C. The blue line shows the data and the red line indicates the fitted model. In this example we chose a ROI in the visual cortex (AAL atlas ROI labeled “Occipital_Mid_R”).

**Figure 3:**
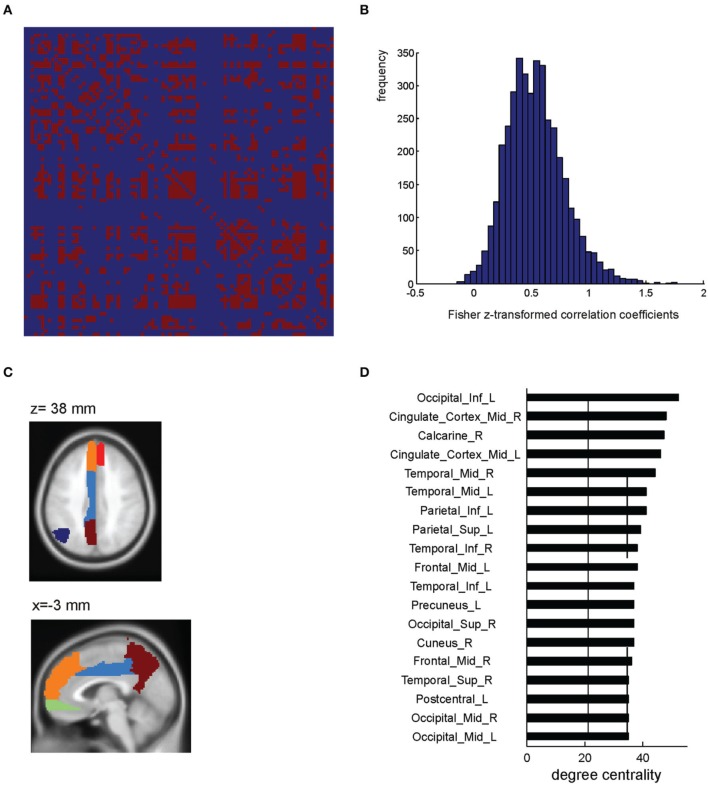
**ROI-based network analysis (single subject level)**. **(A)** Single subject network matrix using the AAL parcellation (Tzourio-Mazoyer et al., 2002). An absolute threshold of *w* > 0.7 was applied to binarize the matrix. **(B)** Distribution of Fisher z-transformed correlation coefficients. **(C)** AAL atlas ROIs showing a high functional connectivity to the precuneus. **(D)** Regions showing a high degree centrality, i.e., one standard deviation above the mean (indicated by the solid and dashed lines).

In order to illustrate the ROI-based network analyses which can be performed using BASCO, we applied a brain parcellation according to the AAL atlas (excluding the cerebellum) to the data and extracted the mean beta-series for each region. We selected only trials and their corresponding beta-values which are related to emotional stimuli and calculated the network matrix correlating all 90 regional beta-series. This resulted in a 90 × 90 network matrix which is shown in Figure 3A (single subject). For the purpose of this demonstration the network matrix was thresholded keeping only weights, i.e., correlation coefficients, larger than *w* = 0.7. All remaining weights were set to one. This resulted in a binary, undirected connectivity matrix. Figure 3B shows all entries of the network matrix before a threshold was applied. Note, that the correlation coefficients were Fisher z-transformed. BASCO offers the functionality to conveniently visualize all brain regions which are strongly connected to a given seed-region. This is illustrated in Figure 3C. The left precuneus was chosen as a seed region. Evaluating the connectivity matrix, we found that the precuneus is connected to the left angular gyrus, left cingulate cortex and the medial frontal cortex (bilateral). The degree centrality is a simple metric for characterizing the role of individual nodes within a network. It refers to the number of connections to other nodes in the network. Nodes with a high degree centrality can be regarded as hubs important for network integration. Figure 3D shows the network nodes with a degree centrality one standard deviation above the mean.

**Figure 4:**
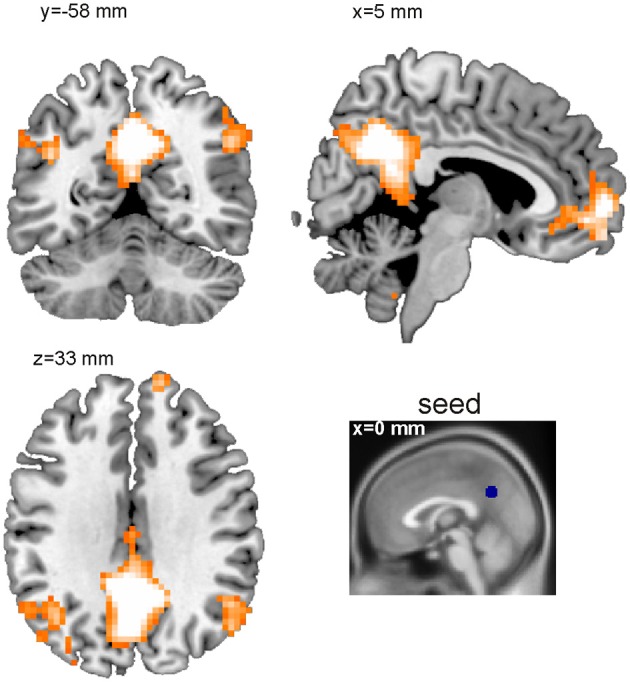
**Seed-based functional connectivity analysis (single subject level)**. Shown is a thresholded functional connectivity map (*z* > 0.7). The seed was placed in the precuneus (MNI c.o.m. coordinates 0, −57, 33 mm).

The BASCO toolbox also allows performing seed-based functional connectivity analyses, where the mean regional beta-series in a seed region is correlated with all voxel beta-series. This approach is useful in a hypothesis driven analysis where a priori seed regions can be derived. Figure 4 depicts a single-subject connectivity map based on trials with emotional content. The seed was placed in the precuneus (sphere with a radius of *r* = 6 mm; MNI coordinates of ROI center: 0, −57, 33 mm). For visualization, the resulting map of voxel-wise correlation coefficients was thresholded at *z* > 0.7. We found the precuneus to be functionally connected to the angular gyrus and the medial frontal cortex. This is in good agreement with the ROI-based analysis shown in Figure 3C. One notable difference is the stronger connectivity to the middle cingulate gyrus observed in the ROI-based but not in the voxel-based analysis. This might be explained by a bias introduced due to the rather large and functionally inhomogeneous AAL atlas ROIs (Göttlich et al., 2013). If applicable, BASCO also allows restricting the analysis to certain brain regions using a mask to reduce the number of statistical tests.

**Figure 5:**
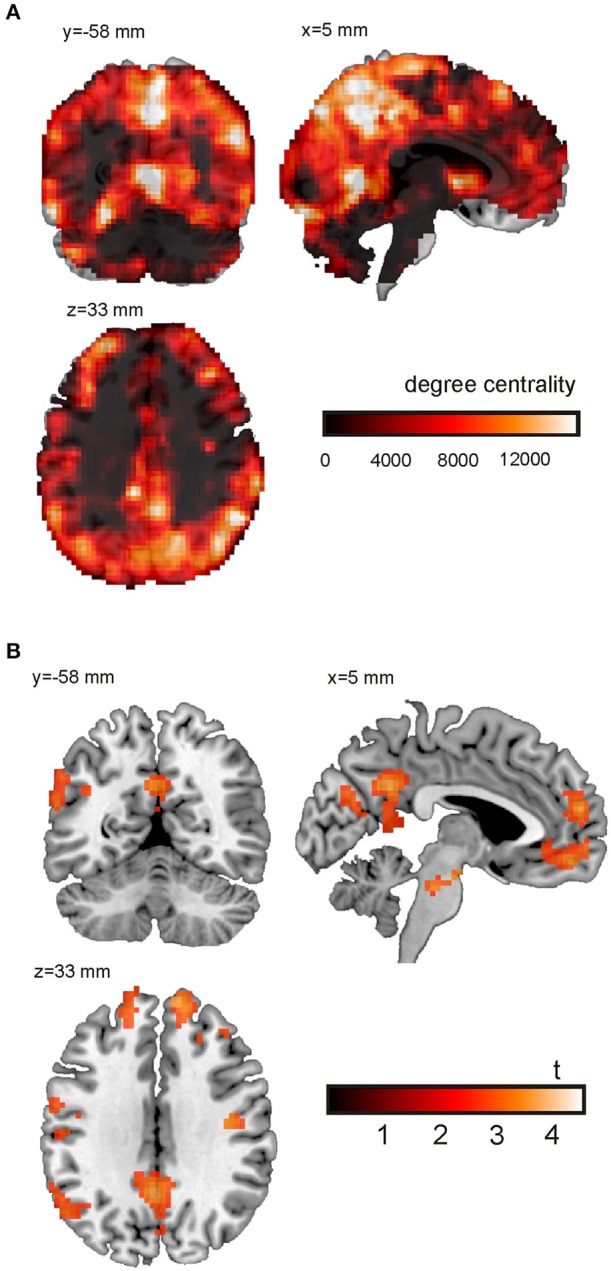
**Voxel-wise degree centrality**. **(A)** Degree centrality map for the emotional condition (single subject). **(B)** Main effect of emotional vs. neutral content on the degree centrality. Shown is the result of an analysis based on the degree centrality maps of 26 subjects. For the purpose of this illustration, uncorrected data (*p* < 0.005; cluster size *k* > 100; **Table 1**) are presented.

Finally, Figure 5A shows a single subject voxel-level degree centrality map derived from trials with emotional content. The tutorial data was resampled to 3 × 3 × 3 mm^3^ resulting in 70,000 voxels for whole brain analyses. The network matrix contains 2.45 billion unique entries. The degree centrality was evaluated using a mask covering the whole brain and applying a threshold of *w* = 0.6 to the correlation coefficients. As expected, ventricles and white matter showed a low degree centrality. In general, gray matter shows a higher degree centrality. The highest degree centrality values are observed in the precuneus, angular gyrus, anterior insula and fusiform gyrus.

The main effect of emotional vs. neutral content on the degree centrality is presented in Figure 5B and Table 1. Shown is the result of an analysis based on the degree centrality maps of 26 subjects (21 women; age = 23.0 ± 3.3 years). We set up a 2 × 2 factorial GLM on the second level (group level) with the factors emotion (two conditions: negative or neutral) and social (two conditions: one person only or two persons interacting). The design matrix included both main effects and interactions. The degree centrality maps related to the following four conditions served as input to the model fit: emotional/single, emotional/social, neutral/single and neutral/social. For the purpose of this illustration, uncorrected data (*p* < 0.005; cluster size *k* > 100) are presented. The 0.05 FWE corrected critical cluster size was *k* = 304. We found a higher degree centrality in the precuneus, angular gyrus, temporo-parietal junction, medial prefrontal cortex (PFC), lateral PFC and the anterior insula.

**Table 1:** **Main effect of emotion on voxel-wise degree centrality**.

**Anatomical region**		***p***	**Size**	***p***	**Peak**	**MNI coordinates [mm]**		
		**(FWE)**		**(unc.)**	***T***	***x***	***y***	***z***
Temporal pole	L	0.013	424	0.0011	4.52	−48	9	−33
Precentral gyrus	L				3.69	−60	6	24
Postcentral gyrus	L				3.64	−54	−24	27
Midbrain	R	0.125	226	0.0116	4.25	3	−24	−6
Midbrain	R				4.00	6	−30	−21
Thalamus	R				3.68	15	−3	6
Precentral gyrus	R	0.047	307	0.0042	4.23	45	−12	30
Superior temporal gyrus	R				3.95	54	−12	15
Superior temporal gyrus	R				3.84	42	−27	−3
Posterior cingulate cortex	L	0.049	304	0.0044	3.73	−3	−51	30
Precuneus	L				3.10	−3	−75	24
Precuneus	L				3.07	3	−51	21
Superior frontal gyrus	R	0.002	595	0.0002	3.71	15	54	36
Middle frontal gyrus	R				3.7	39	39	15
Inferior frontal gyrus	R				3.69	42	27	9
Middle frontal gyrus	L	0.213	183	0.0208	3.62	−45	48	−9
Inferior orbito-frontal gyrus	L				3.48	−42	33	−15
Middle frontal gyrus	L				3.25	−39	48	3
Middle frontal gyrus	L	0.41	130	0.0458	3.35	−27	48	24
Superior frontal gyrus	L				3.31	−21	54	27
Superior frontal gyrus	L				3.29	−18	51	36
Supramarginal gyrus	L	0.512	111	0.0623	3.23	−63	−51	27
Angular gyrus	L				3.18	−51	−63	30
Superior temporal gyrus	L				3.16	−57	−57	21

## Discussion and conclusions

Frequently, brain regions which are co-activated during a given task or stimulus are referred to as a network. However, a network is not fully characterized by its nodes, but can only be fully understood through the links between them, so-called edges, which reflect the interdependencies within the network in the sense of functional integration. The BASCO toolbox allows establishing the functional connectivity between network nodes from slow event-related fMRI data. This extends mass-univariate activation and resting-state functional connectivity analyses and provides new insights into the interplay of different brain regions processing a certain stimulus or executing a task. In contrast to DCM analyses, this approach is suitable for data-driven whole-brain network analyses including a large number of network nodes. The disadvantage compared to DCM is that no information on the causality can be inferred.

A few limitations apply to the presented analysis approach in BASCO. The method to introduce a regressor in the GLM for each single trial is particularly suitable for slow event-related designs with stimulus durations longer than the fMRI repetition time. Under these conditions one can expect a stable model fit. Fitting fast event-related data with rather short stimulus durations (< TR) is problematic as this approach is susceptible to fit noise. Another limitation is related to the number of trials for a given experimental condition. The variance of the Fisher z-transformed correlation coefficients is proportional to the number of trials: σ^2^ = (*N*−3)^−1^. This equation can be used to judge the sensitivity of an experiment given an expected effect size and the number of subjects included in the study. While there is no universally valid recommendation for the minimum number of trials, an analysis based on *N* > 20 trials per condition (length of β-series) is most likely limited by other factors, i.e., the number of subjects and/or the between subject variability.

The number of voxels per ROI and the ROI homogeneity are crucial parameters given a certain parcellation. Averaging voxel β-series of a brain region which is functionally inhomogeneous may not be valid and may lead to biased results, i.e., network properties (e.g., the degree centrality) may depend on the parcellation scheme. This stresses the advantage of using parcellation schemes with finer granularity or to switch to voxel-based approaches to overcome these limitations. The disadvantage of voxel-based approaches, however, is the higher number of statistical tests which have to be performed and corrected for. Furthermore, a drawback of the voxel-by-voxel whole brain network approaches is that the connectivity matrix is larger by several orders of magnitude compared to ROI-based analyses, which is problematic for complex network measures. However, simple network measures such as the degree centrality can be evaluated efficiently at voxel-level providing a high spatial resolution. In the future, the toolbox could be extended by implementing more complex network metrics for voxel-wise whole-brain network analyses, e.g., the clustering and the characteristic path length. The degree centrality map is evaluated for a given threshold which is applied to the connectivity matrix. We strongly recommend checking if the results of an analysis critically depend on the choice of the threshold. The default threshold is 0.25. One option is to repeat the analysis at least twice choosing 0.2 and 0.3 as a threshold and to check if the results can be reproduced.

The GUI and the multitude of implemented analysis approaches in BASCO hopefully lower the threshold to perform functional connectivity analyses of event-related fMRI data. It may thus contribute to a broader application of this method which offers a new perspective to investigate brain function and extends and compliments standard univariate and resting-state analyses.

## Funding

This work was supported through intramural funding of the University of Lübeck (SPP4-C1 to UK).

### Conflict of interest statement

The authors declare that the research was conducted in the absence of any commercial or financial relationships that could be construed as a potential conflict of interest.
